# Prescribing Appropriateness and Clinical Outcomes in Hospitalized Adults with COVID-19 in Jazan, Saudi Arabia: A Retrospective Cohort Study

**DOI:** 10.3390/healthcare14193328

**Published:** 2026-10-06

**Authors:** Mohammed H. Abutaleb, Mohammed Y. Asiri, Santhosh J. Menachery, Ahmed A. Albarraq, Hafiz A. Makeen

**Affiliations:** 1 Pharmaceutical Care Department, King Fahd Central Hospital, Jazan 82666, Saudi Arabia; 2 Prince Mansour Military Hospital, Taif 26526, Saudi Arabia; 3 Pharmacy Practice Research Unit, Department of Pharmacy Practice, College of Pharmacy, Jazan University, Jazan 45142, Saudi Arabia

**Keywords:** COVID-19, prescribing appropriateness, Medication Appropriateness Index, clinical outcomes, hospitalization, Saudi Arabia, Jazan, pharmacotherapy stewardship

## Abstract

**Highlights:**

**What are the main findings?**

- Prescribing followed contemporaneous national guidance where the evidence was settled (anticoagulants, corticosteroids) but diverged for agents whose evidence remained contested.
- Diabetes, hypertension, and cardiac disease were each associated with approximately double the unadjusted odds of in-hospital death, and patients who died were, on average, 15.8 years older than survivors.

**What are the implications of the main findings?**

- Inappropriateness arose predominantly within the duration and indication domains, favoring automatic stop orders and indication-linked order sets over dose-checking systems, with periodic MAI-based audit and pharmacist-led stewardship as practical means of sustaining guideline concordance.
- Risk stratification of high cardiometabolic-burden populations should be a standing element of pandemic preparedness.

**Abstract:**

**Background:** The COVID-19 pandemic required repeated revision of national treatment protocols; evaluating prescribing against the guidance then in force is central to stewardship. **Objective:** To characterize clinical outcomes of adults hospitalized with COVID-19 in Jazan, Saudi Arabia, and to assess prescribing appropriateness using the Medication Appropriateness Index (MAI) benchmarked against contemporaneous Ministry of Health (MOH) guidance. **Methods:** Single-center retrospective cohort study of all adults (aged 18 years or over) admitted with COVID-19 between 1 March 2020 and 28 February 2021. Two trained clinical pharmacists independently applied the MAI to 1018 prescriptions. All associations were examined in univariable, unadjusted analyses. **Results:** Hypertension (44.1%), diabetes (38.7%), end-stage renal disease (13.7%), and cardiac disease (12.4%) were prevalent. In-hospital mortality was 26.8% (121/451 patients with a determinate outcome). Diabetes (OR 2.02, 95% CI 1.32–3.08), hypertension (OR 1.92, 95% CI 1.26–2.93), and cardiac disease (OR 2.04, 95% CI 1.15–3.64) were each associated with higher unadjusted odds of death; patients who died were on average 15.8 years older (*p* < 0.0001). Overall, 936 of 1018 prescriptions (91.9%) were appropriate; rates ranged from 99.4% (311/313) for enoxaparin to 42.9% (6/14) for hydroxychloroquine, the latter based on a small number of prescriptions. Inappropriate prescriptions were flagged mainly in the duration (82.9%) and indication (70.7%) domains, and receipt of at least one inappropriate prescription was not associated with mortality (OR 0.59, 95% CI 0.31–1.12; *p* = 0.10). **Conclusions:** Anticoagulants and corticosteroids were largely prescribed in line with contemporaneous MOH guidance; several repurposed agents were not. These findings support an MAI-based audit against the guideline version in force as a tool for quality control.

## 1. Introduction

The coronavirus disease 2019 (COVID-19) pandemic, caused by severe acute respiratory syndrome coronavirus 2 (SARS-CoV-2), represents one of the most consequential global health emergencies of the twenty-first century. First identified in Wuhan, China, in December 2019, the virus was rapidly linked to clusters of severe pneumonia and subsequently classified as a novel β-coronavirus within the subgenus Sarbecovirus. Its rapid global dissemination prompted the World Health Organization (WHO) to declare a Public Health Emergency of International Concern in January 2020, followed by the formal designation of a global pandemic in March 2020 [1].

SARS-CoV-2 is transmitted primarily via respiratory droplets and aerosols [2]. The incubation period ranges from 2 to 14 days, and clinical manifestations span a spectrum from asymptomatic infection to critical illness [3], with severe cases progressing to pneumonia, acute respiratory distress syndrome (ARDS), septic shock, and multi-organ failure [4]. Lymphocytopenia and elevated C-reactive protein (CRP), ferritin, D-dimer, and hepatic enzymes are frequent in severe disease and serve as prognostic indicators [5]. Clinical outcomes are strongly influenced by demographic and comorbid risk factors [6]. Advanced age remains the most consistent predictor of mortality, whilst male sex has been associated with worse outcomes, potentially reflecting immunological, hormonal, and behavioral differences [6]. Chronic conditions, including diabetes mellitus, hypertension, cardiovascular disease, chronic kidney disease, and obesity, significantly increase the risk of hospitalization, intensive care unit (ICU) admission, and death [7].

The therapeutic landscape for COVID-19 evolved rapidly in response to accumulating clinical evidence. Initial treatment strategies were largely empirical, relying on supportive care and off-label use of repurposed agents. As randomized controlled trials (RCTs) and large-scale observational studies emerged, clinical guidelines were revised accordingly. RECOVERY established a significant mortality benefit for dexamethasone in patients requiring supplemental oxygen or ventilation [8], and trials of anticoagulation with low-molecular-weight heparin (LMWH) informed thromboprophylaxis against COVID-19-associated coagulopathy [9,10,11]. Remdesivir showed modest reductions in time to recovery [12], whilst favipiravir yielded inconsistent results [13], and tocilizumab was introduced to attenuate cytokine storm with initially variable outcomes [14,15]. Conversely, hydroxychloroquine and lopinavir/ritonavir, adopted early on preliminary mechanistic and observational grounds, were de-implemented following the WHO Solidarity and TOGETHER trials [16,17].

The sequence in which this evidence emerged determines how prescribing in any given period should be judged. The present study period, 1 March 2020 to 28 February 2021, covers the first year of the pandemic and precedes both the global peak in cases and the global peak in deaths. Through much of this window, several agents later shown ineffective were still permitted by national and international guidance, whereas remdesivir was widely regarded as the most promising available antiviral. Assessment of prescribing quality in this period must therefore be anchored to the guidance in force at the moment of prescribing rather than to subsequent evidence.

Prescribing appropriateness became a central concern throughout the pandemic. In this study, it is defined as the concordance of medication use with the guidance in force at the time of prescribing. The Medication Appropriateness Index (MAI), a validated clinimetric measure covering ten domains including indication, effectiveness, dosage, directions, interactions, duplication, duration, and cost, is widely used to assess prescribing quality in hospital settings [18,19]. Its application to COVID-19 has been limited, partly because national guidelines were revised repeatedly rather than remaining static, complicating the definition of a fixed appropriateness standard. This difficulty is surmountable through version-specific benchmarking, and the MAI retains value as a structured, reproducible instrument for retrospective evaluation. International studies have consistently identified notable rates of inappropriate prescribing among hospitalized COVID-19 patients, including antibiotic overuse and use of agents of uncertain clinical benefit [20,21,22].

In Saudi Arabia, the Ministry of Health (MOH) revised COVID-19 guidelines frequently between 2020 and 2022 to incorporate emerging evidence [23,24,25]. The extent to which prescribing aligned with these contemporaneous recommendations has not been systematically evaluated across regions; published studies have focused predominantly on urban centers such as Riyadh, Jeddah, and Dammam [24,26,27,28,29,30], leaving peripheral regions underrepresented. The Jazan region, located in south-western Saudi Arabia, presents a distinct and clinically relevant epidemiological profile. Despite a relatively young population, it exhibits a disproportionately high prevalence of diabetes and hypertension, comorbidities known to amplify COVID-19 severity and mortality [31,32]. Limited data exist on outcomes and prescribing in this high-risk population, a gap of consequence given the potential for inappropriate prescribing to increase harm, inflate costs, and undermine national treatment strategies.

This study therefore had two primary objectives: (1) to characterize the epidemiological profile and short-term clinical outcomes (in-hospital mortality and length of hospital stay [LOS]) of adults hospitalized with laboratory-confirmed COVID-19 in Jazan; and (2) to systematically evaluate the appropriateness of COVID-19 pharmacotherapy using the MAI, benchmarked against the Saudi MOH protocol contemporaneous with each admission. A secondary, exploratory objective was to examine whether prescribing appropriateness was associated with clinical outcome, recognizing the methodological constraints on such an analysis in a retrospective dataset.

## 2. Materials and Methods

### 2.1. Study Design and Setting

A single-center retrospective observational cohort study was conducted at a tertiary care hospital in Jazan Province, Kingdom of Saudi Arabia, from 1 March 2020 to 28 February 2021. The institution served as the principal referral center for severe COVID-19 in the province, a case-mix consideration relevant to interpretation of the observed mortality (Section 4.2). The study is reported in accordance with the Strengthening the Reporting of Observational Studies in Epidemiology (STROBE) statement [33].

### 2.2. Study Population and Patient Selection

Adults aged 18 years or over admitted with SARS-CoV-2 infection confirmed by real-time reverse transcription polymerase chain reaction (RT-PCR) were eligible, regardless of sex; patients younger than 18 years were not eligible, and none were included. RT-PCR testing of respiratory specimens was performed as part of routine clinical care by the hospital microbiology laboratory; the investigators used no commercial reagents, kits or devices for the purposes of this study. Patients were to be excluded if RT-PCR confirmation was absent or if the record was insufficient to determine outcome or medication exposure.

**Figure 1:**
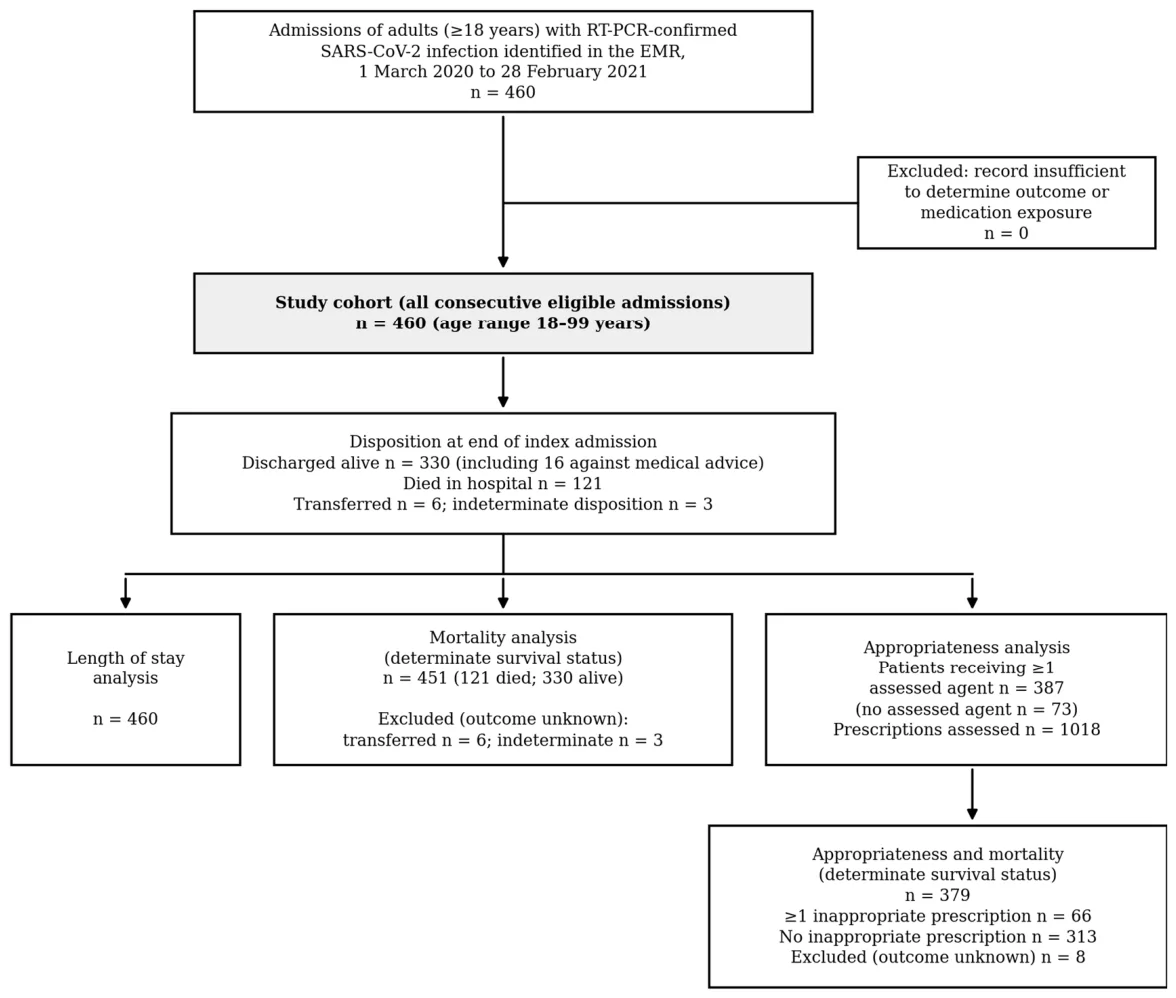
Flow of patients through the study, showing the number included in each analysis and the reasons for exclusion from that analysis. EMR, electronic medical record; LOS, length of hospital stay; RT-PCR, real-time reverse transcription polymerase chain reaction.

A total of 460 RT-PCR-confirmed admissions were identified in the hospital’s electronic medical record (EMR) system, all of which met the eligibility criteria and were retained; none was excluded. The analytic cohort therefore comprises all consecutive eligible adult admissions during the study period, not a selected sample. Patient flow from identification to each analytic subset is shown in Figure 1.

### 2.3. Sample Size

At study initiation, the Saudi MOH had reported 284,226 cumulative confirmed COVID-19 cases nationally, of which 34,082 were active (6 August 2020) [34]. Cochran’s formula [35], applied with a 95% confidence level, 5% margin of error, a conservative prevalence estimate of 50% and finite population correction for the number of active cases, gave a minimum of 380 patients, or 400 with a 5% contingency. Because all eligible admissions were included, the final sample of 460 exceeded this threshold; the calculation confirmed the adequacy of the available cohort rather than determining a sampling fraction.

### 2.4. Data Collection

Data were extracted from EMRs using standardized abstraction forms developed in Microsoft Excel for Mac, version 16 (Microsoft Corp., Redmond, WA, USA), which were piloted before use to minimize extraction variability. Variables recorded included demographic characteristics (age, sex, and nationality); comorbidities; vital signs at admission; mechanical ventilation status and duration; laboratory and radiological findings; pharmacological data (drug name, dose, route, frequency, duration, and cost); and clinical outcomes (discharged alive, in-hospital death, or transfer to another facility).

### 2.5. Study Outcomes

The study had two co-primary outcome domains, corresponding to the two elements of the title. The first comprised the clinical outcomes of hospitalization: in-hospital mortality, a binary outcome defined restrictively as a death recorded during the index admission, and LOS, a continuous outcome in days from admission to discharge, death or transfer. Patients who left against medical advice were counted as discharged alive, since they left the hospital living; their post-discharge outcome was not ascertained and is not claimed. Patients whose disposition could not be determined were classified separately. Neither group was counted as deceased. The second primary domain was prescribing appropriateness, defined as the proportion of prescriptions classified appropriate by the MAI against the MOH guideline version in force on the admission date.

Secondary outcomes were transferred to another facility as a disposition category; associations of demographic and comorbid characteristics with each primary clinical outcome; and the exploratory relationship between appropriateness and outcome (Section 3.7). Mechanical ventilation status, laboratory parameters, and radiological findings were collected to characterize the cohort and are descriptive only; they were not treated as outcomes.

### 2.6. Medication Appropriateness Assessment

Prescribing appropriateness was evaluated using the MAI, a validated instrument developed by Hanlon et al., which assesses ten domains: indication, effectiveness, dosage, correct directions, practical directions, drug–drug interactions, drug–disease interactions, unnecessary duplication, duration, and cost [18,19]. The cost domain was excluded because medications are supplied free of charge under the publicly funded national system, so cost did not influence prescribing decisions and was not clinically relevant to appropriateness in this context. The nine remaining domains give a cumulative range of 0–17 per prescription. Each was rated on the standard three-point scale (A = appropriate, B = marginally appropriate, C = inappropriate) using the instrument’s explicit criteria, with B or C ratings assigned the corresponding Hanlon weight and summed. Consistent with validated application, a cumulative score of 3 or above indicated clinically meaningful inappropriateness [19], a threshold applied uniformly to all 1018 prescriptions.

Two clinical pharmacists experienced in infectious diseases independently assessed every prescription after structured training. Training, led by a senior clinical pharmacist (MHA), covered Hanlon’s operational definitions and explicit criteria for each domain, the weighting scheme and the guideline-version rules described below; decision rules agreed during training were recorded in a written scoring guide. Before definitive scoring, both reviewers independently scored a pilot sample of 50 prescriptions, compared their ratings domain by domain and harmonized the application of the criteria. Each disagreement during definitive scoring was documented (prescription, domain, each reviewer’s rating and the agreed rating) and resolved through structured consensus discussion; unresolved cases were referred to a third clinical pharmacist, whose decision was final (9 prescriptions resolved by consensus and 3 by adjudication). Inter-rater agreement was not calculated because only post-consensus ratings were retained in the analytic dataset, so a chance-corrected statistic such as Cohen’s κ [36] cannot be computed retrospectively. This is acknowledged as a limitation (Section 4.5).

Each prescription was benchmarked against the version of the Saudi MOH COVID-19 clinical management guideline that was active at the time of the patient’s admission [37]. The protocol was revised repeatedly during the study period, and the versions applied included version 1.1 (19 March 2020) [25], version 1.2 (5 April 2020) [38], version 1.8 (2 June 2020) [39], and version 2.0 (17 June 2020) [40]. Recommendations applied included hydroxychloroquine 400 mg twice daily on day 1, then 200 mg twice daily for 5 to 7 days (versions 1.2 to 2.0) [38,39,40], and favipiravir 1800 mg twice daily on day 1, then 800 mg twice daily for 7 to 10 days (version 2.0) [40,41]; by January 2021, favipiravir or remdesivir was recommended for severe and critical disease, dexamethasone was the preferred corticosteroid, and hydroxychloroquine was limited to early treatment of mild disease in outpatients [42]. Archived guideline versions were reviewed systematically to align each prescribing decision with the contemporaneous recommendations and the pharmacotherapies available during the study period. Appropriateness was therefore judged solely against recommendations available to the prescriber at the time, not against evidence that emerged subsequently. Where a version permitted an agent under specified conditions, a prescription was classified as appropriate only if documentation confirmed those conditions were met.

### 2.7. Statistical Analysis

Data were analyzed using IBM SPSS Statistics, version 22.0 (IBM Corp., Armonk, NY, USA). Categorical variables are summarized as frequencies and percentages; continuous variables are reported as means ± standard deviation (SD) or medians with interquartile ranges (IQR) according to distribution. Categorical comparisons used Pearson’s chi-square test, with Fisher’s exact test where any expected cell count was below five; two-group comparisons of continuous variables used the Mann–Whitney U test and multi-group comparisons the Kruskal–Wallis test, except age by outcome, compared by one-way ANOVA. All tests were two-tailed, with statistical significance set at *p* < 0.05.

To convey the magnitude and precision of associations with mortality, unadjusted odds ratios (ORs) with 95% CIs were calculated for in-hospital death by the Woolf logit method [43], restricted to the 451 patients with a determinate survival status at the end of the index admission (121 deaths, 330 discharged alive); the 6 transferred and 3 indeterminate patients were excluded because their eventual outcome is unknown. LOS analyses used the full cohort (*n* = 460) and appropriateness analyses all 1018 prescriptions.

No multivariable regression models were fitted; the analysis is univariable throughout. This was deliberate, because validated severity-of-illness measures, namely the Sequential Organ Failure Assessment (SOFA) score [44] and the Acute Physiology and Chronic Health Evaluation II (APACHE II) score [45], could not be reconstructed, as their constituent parameters were not consistently documented in early pandemic records. Baseline severity is the dominant confounder of every association examined, and a model omitting it would yield adjusted estimates carrying unwarranted precision and an unjustified implication of independent effect. All associations are therefore unadjusted and are described as such throughout; the terms “independently associated”, “predictor” and “determinant” are not used. 

Missing data were negligible; no values were missing for age, sex, nationality, LOS, diabetes, hypertension, cardiac disease, or renal disease, nor for any MAI domain rating across the 1018 assessed prescriptions. Discharge disposition could not be determined for 3 patients (0.7%), who are retained in the cohort description and LOS analyses but excluded from mortality comparisons. Vital signs, laboratory, and radiological variables were documented inconsistently in early pandemic records; they were used to describe the cohort only and were not entered into any comparison. Complete-case analysis was used; imputation was not required.

### 2.8. Ethical Considerations

The study was approved by the Research Ethics Committee of the Directorate of Health Affairs, Jazan (IRB No. 2117, approved 1 March 2021). Data were anonymized prior to analysis, and access was restricted to authorized study personnel. Individual consent was waived owing to the retrospective design and the use of anonymized records, consistent with national regulations and institutional policy.

## 3. Results

### 3.1. Cohort Characteristics

A total of 460 adults (aged 18 years or over) with RT-PCR-confirmed COVID-19 were included (Figure 1). The mean age was 51.0 ± 20.2 years (range 18–99); most were Saudi (82.8%) and male (58.9%). The median LOS was 7 days (IQR: 4–14; range: 1–215 days). The most prevalent comorbidities were hypertension (44.1%, *n* = 203), diabetes mellitus (38.7%, *n* = 178), end-stage renal disease (ESRD; 13.7%, *n* = 63), and cardiac disease (12.4%, *n* = 57). Detailed baseline characteristics are presented in Table 1.

### 3.2. Clinical Outcomes and Associations with Comorbidity

Overall clinical outcomes and their associations with key comorbidities are presented in Table 2. Among the 460 patients, 330 (71.7%) were discharged alive (including 16 who left against medical advice), 121 (26.3%) died in hospital, 6 (1.3%) were transferred to another facility, and 3 (0.7%) had an indeterminate disposition. Among the 451 patients with a determinate survival status, the denominator used for all comparisons below, in-hospital mortality was 26.8% (121/451).

Diabetes (OR 2.02, 95% CI 1.32–3.08; *p* = 0.001), hypertension (OR 1.92, 95% CI 1.26–2.93; *p* = 0.002), and cardiac disease (OR 2.04, 95% CI 1.15–3.64; *p* = 0.014) were each associated with approximately double the unadjusted odds of death; the consistency of the three estimates is more informative than any one alone. Sex, nationality, and renal disease showed no significant association, and the AKI estimate rests on eleven patients with a correspondingly uninformative interval (Table 2). All odds ratios are unadjusted and describe association, not independent effect; they are adjusted neither for age, itself strongly associated with mortality (Section 3.4), nor for baseline severity, which could not be quantified.

### 3.3. Length of Hospital Stay

In unadjusted comparisons, median LOS was longer in men than women (8 vs. 6 days; *p* = 0.006), in non-Saudi than Saudi patients (9 vs. 7; *p* = 0.008), and in patients with diabetes (9 vs. 6; *p* < 0.001) or hypertension (9 vs. 6; *p* < 0.0001). AKI had the longest median LOS of any renal subgroup (11 days), though the category overall was not significant (*p* = 0.28); cardiac disease was not associated with LOS (*p* = 0.50) (Table 3).

### 3.4. Age, LOS, and Clinical Outcome

In unadjusted comparisons, age was strongly associated with clinical outcome (*p* < 0.0001). Patients who died were on average 15.8 years older than those discharged alive (62.6 ± 17.2 vs. 46.9 ± 19.6 years; *p* < 0.0001) and had a longer median LOS (11 vs. 6 days; *p* < 0.0001) (Table 4).

### 3.5. Prescribing Appropriateness by Agent

Of 1018 prescriptions, 936 (91.9%) were appropriate and 82 (8.1%) inappropriate. Guideline concordance was high for enoxaparin (99.4%), favipiravir (94.9%), ribavirin (92.9%), and dexamethasone (92.0%); lower for tocilizumab (79.6%) and lopinavir/ritonavir (71.9%); and lowest for the two least-used agents, remdesivir (3/5, 60.0%), and hydroxychloroquine (6/14, 42.9%) (Table 5).

These last two estimates warrant particular caution: with five and fourteen prescriptions, a single reclassification alters the rate by 20.0 and 7.1 percentage points. They are descriptive observations for the prescriptions examined, not stable estimates of institutional prescribing quality, and do not support generalization.

### 3.6. Distribution of MAI Domains

Across the 1018 prescriptions, 478 (47.0%) received a less-than fully appropriate rating in at least one domain, giving 596 domain-level ratings (Table 6). Two patterns emerge, differing by the question asked. Across all prescriptions, the most frequently flagged domains were dosage (19.4%) and duration (18.0%); the dosage figure is driven by enoxaparin, for which 147 of 313 prescriptions received a marginal weight-based rating that did not reach the inappropriateness threshold. Among the 82 inappropriate prescriptions, however, duration was implicated in 82.9% and indication in 70.7%, while dosage fell to 15.9%. Clinically meaningful inappropriateness was therefore driven by whether an agent was indicated and how long it continued, not by errors of dose or administration. Patterns were drug-specific: indication ratings were concentrated in tocilizumab (55 of 142), drug–drug interaction ratings in favipiravir (55 of 297) and lopinavir/ritonavir (23 of 32).

This disaggregation is directly usable by pharmacy and therapeutics committees, pharmacovigilance programs and internal quality control, since it identifies which element of the prescribing decision requires intervention: here, point-of-order decision support and automatic stop orders rather than dose-checking systems.

### 3.7. Prescribing Appropriateness in Relation to Clinical Outcome

Prescription records were linked to individual patients, yielding 387 patients who received at least one assessed agent, of whom 66 (17.1%) received at least one inappropriate prescription. Among the 379 with determinate survival status, mortality was 21.2% (14/66) in exposed patients versus 31.3% (98/313) in unexposed (OR 0.59, 95% CI 0.31–1.12; *p* = 0.10), and median LOS did not differ materially (6 vs. 8 days; *p* = 0.41). Inappropriate prescribing was therefore not associated with higher mortality or longer stay in this unadjusted analysis.

This null result is not evidence that appropriateness is unimportant, and the point estimate below unity does not indicate benefit from inappropriate prescribing. Two features preclude a causal reading in either direction. Appropriateness was assessed per prescription rather than per patient, and exposed patients received more assessed medications (mean 3.27 vs. 2.50), so exposure is partly a marker of treatment intensity. Confounding by indication also operates in both directions: exposed patients were more likely to have received tocilizumab (57.6% vs. 32.1%), reserved for the severely ill, yet also agents such as hydroxychloroquine used earlier and in milder illness. Without a validated severity score these opposing pressures cannot be separated, and the result is interpreted no further.

## 4. Discussion

This retrospective cohort study examined clinical outcomes and prescribing appropriateness among 460 adults hospitalized with RT-PCR-confirmed COVID-19 in the Jazan region during the first year of the pandemic. Because the study was retrospective, single-center, and unadjusted for baseline disease severity, all reported relationships represent associations observed within this cohort and do not establish causation. In particular, the absence of an observed association between inappropriate prescribing and mortality should not be interpreted as evidence that inappropriate prescribing is clinically benign, given the unadjusted nature of the analysis and the potential for confounding by illness severity and treatment indication.

### 4.1. Epidemiological Profile and Comorbidity Burden

The cohort carried a substantial burden of chronic non-communicable disease. Diabetes mellitus (38.7%), hypertension (44.1%), and ESRD (13.7%) were markedly prevalent, exceeding rates reported in many national and international COVID-19 cohorts [28,32,46,47]. Diabetes demonstrated a statistically significant unadjusted association with in-hospital mortality (OR 2.02), consistent with meta-analytic evidence of a two- to three-fold increase in COVID-19 mortality among individuals with diabetes [47], although the biological mechanisms proposed in the literature [48] were not examined in the present study. Hypertension (OR 1.92) and cardiac disease (OR 2.04) showed comparable associations, and diabetes and hypertension, but not cardiac disease, were also associated with prolonged hospitalization.

These comorbidities are strongly intercorrelated with one another and with age, and none of the estimates are adjusted. The data support the observation that patients with a heavier cardiometabolic burden died more often and stayed longer; they do not establish the independent contribution of any one condition.

### 4.2. In-Hospital Mortality in Context

The in-hospital mortality of 26.8% (121/451 patients with a determinate outcome; 26.3% of the full cohort of 460) is higher than many contemporaneous series [27,49] and requires contextualization rather than direct comparison. It counts only deaths recorded during the index admission: the 16 patients who left against medical advice left alive and are counted as discharges, and the 6 transferred and 3 indeterminate patients are excluded from the denominator because their eventual outcome is unknown. Estimates absorbing such patients into the numerator are correspondingly higher and not comparable.

Four considerations, none of which could be tested with these data, bear on the figure. The denominator is restricted to hospitalized patients at the provincial referral center for severe COVID-19, where case-fatality is systematically higher than from population-based denominators [50]. The study period precedes both the global case and death peaks and predates several mortality-reducing treatments and vaccination. The cohort carried an unusually heavy comorbidity load, each component of which was associated with death in unadjusted analysis. Finally, pandemic mortality estimation was affected across settings by heterogeneity in testing intensity, admission thresholds and outcome ascertainment [51]. Together these suggest the observed mortality reflects a severe-case referral denominator, an early-pandemic treatment era and a regionally distinctive comorbidity burden rather than a deficiency of care, although this inference cannot be confirmed without adjustment for baseline severity.

### 4.3. Age and Length of Stay

Age showed the strongest association with mortality: patients who died were on average 15.8 years older than those discharged alive (62.6 vs. 46.9 years; *p* < 0.0001), consistent with the well-established association between older age and COVID-19 mortality in published cohorts [27,28,49,52,53]; the underlying biological mechanisms were not assessed in this study. Older patients also carried more cardiometabolic comorbidity, so the age association cannot be disentangled from those in Section 4.1.

Median LOS of 7 days is broadly consistent with reports for this period [53,54]. Men were hospitalized longer than women (8 vs. 6 days), a difference also reported in other cohorts [55]. Non-Saudi patients stayed longer than Saudi nationals (9 vs. 7 days); the reasons for this difference were not assessed in this study. Diabetes and hypertension were each associated with longer stay, and AKI with the longest stay of any renal subgroup [56], though with eleven patients that observation is fragile. These differences were observed rather than explained: longer stay may reflect severity at presentation, complications, slower recovery or discharge planning, which these data cannot distinguish.

### 4.4. Prescribing Appropriateness in the Context of Evolving Guidance

A principal contribution of this study is its systematic, MAI-based evaluation of COVID-19 prescribing appropriateness, benchmarked against guidance in force at the time of each prescription. Overall, 91.9% of prescriptions were guideline-concordant; high rates for enoxaparin (99.4%), favipiravir (94.9%), and dexamethasone (92.0%) are consistent with rapid local adoption of evolving evidence, including thromboprophylaxis recommendations for COVID-19-associated coagulopathy [11,39,57] and the dexamethasone findings of RECOVERY [8,40].

Lower rates for the repurposed agents hydroxychloroquine (8/14, 57.1% inappropriate) and lopinavir/ritonavir (9/32, 28.1%) are consistent with the global pattern of early empirical adoption followed by de-implementation as negative trial evidence accumulated [16,17,25,28,58]. Inappropriate use of tocilizumab (29/142, 20.4%) arose largely from incomplete documentation of eligibility criteria, namely inflammatory marker thresholds and oxygen requirements, at the time of prescribing [40,59,60], highlighting the critical role of structured clinical documentation and real-time decision-support systems in ensuring guideline-concordant use of high-cost immunomodulatory agents.

Remdesivir (3/5, 60.0%) was scored appropriate more often than hydroxychloroquine (6/14, 42.9%), a difference that may be consistent with the evidentiary positions the two occupied at the time: remdesivir was then widely regarded as the most promising antiviral and governed by explicit eligibility criteria, whereas hydroxychloroquine was an inexpensive repurposed agent adopted broadly and empirically before withdrawal from guidance [16,17,40]. Both estimates rest on very small numbers and are unstable, and no conclusion is drawn about either agent or about the difference between them. Retrospective appropriateness assessment is also vulnerable to hindsight: clinicians acted on the evidence then available under acute pressure, and agents governed by narrow eligibility criteria are mechanically more likely to score inappropriate when documentation does not record that those criteria were met. These ratings therefore describe documented concordance with the protocol in force, not the quality of individual clinical reasoning.

Among prescriptions reaching the inappropriateness threshold, duration (82.9%) and indication (70.7%) were implicated far more often than dosage (15.9%). Prescribing divergence here was predominantly a question of whether a drug was warranted and for how long it continued rather than how it was dosed, a distinction with direct consequences for stewardship design (Section 4.6).

### 4.5. Strengths and Limitations

This study has several notable strengths. The cohort is relatively large (460 patients, 1018 individually assessed prescriptions) from a geographically underrepresented region, comprising all consecutive eligible admissions rather than a selected sample. The use of a validated, dual-reviewer MAI application with systematic benchmarking against contemporaneous guideline versions enhances the methodological rigor and clinical relevance of the prescribing appropriateness assessment. The study period captures the full first year of the pandemic, encompassing both the initial empirical and evidence-refinement phases of therapeutic decision-making.

Several limitations must, however, be acknowledged. The retrospective design depends on the completeness and accuracy of clinical documentation, which may have been affected by the operational pressures of the early pandemic. The single-center setting may limit generalizability, although the cohort’s baseline characteristics are broadly consistent with regional epidemiological data [31]. Most consequentially, validated severity-of-illness scoring tools (e.g., SOFA, APACHE II) could not be calculated due to inconsistent availability of relevant parameters in early pandemic records, limiting the ability to adjust for baseline disease severity and introducing the possibility of residual confounding. All reported associations are therefore unadjusted and should not be interpreted as independent or causal effects. Inter-rater agreement for the MAI could not be quantified because only post-consensus ratings were retained (Section 2.6). Estimates for infrequently prescribed agents (remdesivir, 3/5; hydroxychloroquine, 6/14) are imprecise and exploratory. Furthermore, glycemic control could not be characterized because HbA1c was not consistently available; poorly controlled diabetes therefore could not be distinguished, and the aggregate association may conceal heterogeneity. Finally, long-term post-discharge outcomes and adverse drug events were not assessed; both should be addressed in future pharmacoepidemiological studies in this setting.

### 4.6. Implications for Practice and Future Preparedness

These implications follow from observed associations rather than demonstrated causation. First, prescribing oversight requires an explicit institutional mechanism. The finding that much inappropriateness arose from absent documentation of eligibility rather than evidently unsuitable choices suggests that prescribing quality in emergencies is as much a documentation and process problem as a knowledge problem. Periodic MAI-based audit by pharmacy and therapeutics committees offers a reproducible means of detecting drift, and the domain analysis indicates where to direct it. Because inappropriateness arose predominantly from duration and indication rather than dosing, automatic stop orders, mandatory duration review and indication-linked order sets are likely to address most of it, whereas dose-checking software, a common form of decision support, would address comparatively little. Indication ratings were concentrated in tocilizumab (55 of 142 prescriptions), making it a specific target for pre-authorization. Such oversight depends on institutional and regulatory support: Saudi pharmacists report high willingness to assume expanded roles but identify physician resistance, limited advanced training and the absence of an enabling legal framework as barriers [61].

Second, patients with diabetes, hypertension or established cardiac disease warrant closer monitoring during respiratory pandemics. Each condition was associated with approximately double the unadjusted odds of death, and diabetes and hypertension with longer hospitalization. Given their high prevalence in this population, a comparable epidemic could impose a greater burden in Jazan than national projections anticipate. Risk-stratified admission pathways, earlier escalation thresholds and proactive management of chronic disease between emergencies follow from this.

Third, updated recommendations require faster operational implementation. High appropriateness for dexamethasone and enoxaparin shows that rapid uptake of new evidence is achievable, whereas continued prescribing of agents whose evidence had been withdrawn suggests that de-implementation is slower than adoption. Decision-support systems linked to the current guideline version, with automated deactivation of superseded protocols, would narrow this gap, particularly when combined with pharmacist-led review of prescriptions against the guideline in force, a role for which clinical pharmacists are well placed [61]. Documentation of indication and eligibility should itself be treated as a component of prescribing quality, since it determines whether appropriateness can be assessed at all.

Finally, a prospective or well-characterized retrospective design incorporating a validated severity score at admission, patient-level MAI aggregation, and explicit handling of confounding by indication would allow a valid assessment of whether appropriateness is associated with outcome independently of severity. A multicenter design would provide the exposure numbers needed for stable estimates for infrequently used agents.

## 5. Conclusions

This study provides real-world evidence on therapeutic decision-making during the first year of the COVID-19 pandemic in the Jazan region, documenting both the strengths of rapid evidence integration and the persistence of non-recommended prescribing practices. Among 460 adults, in-hospital mortality was 26.8% (121/451 with a determinate outcome). In unadjusted analyses, older age, diabetes, hypertension, and cardiac disease were associated with in-hospital mortality, and diabetes and hypertension with prolonged hospitalization; these associations do not establish independent or causal effects.

The study also demonstrates that the MAI can be applied to pandemic-era pharmacotherapy through version-specific benchmarking against the guidance in force at the time of prescribing, supporting its use as a practical instrument for internal quality control. Frameworks combining MAI-based audit, improved documentation of indication and pharmacist-led review [62,63] offer a concrete mechanism for maintaining prescribing quality when the next emergency again requires decisions faster than evidence accumulates.

## Figures and Tables

**Table 1:** Demographic and clinical characteristics of adults hospitalized with COVID-19 (*n* = 460).

Variable	*n* (%)
Age, years: mean ± SD	51.0 ± 20.2
Age, years: median (IQR)	53 (37–66)
Male sex	271 (58.9)
Saudi nationality	381 (82.8)
Diabetes mellitus	178 (38.7)
Hypertension	203 (44.1)
Cardiac disease	57 (12.4)
Renal disease: none	377 (82.0)
Renal disease: ESRD	63 (13.7)
Renal disease: AKI	11 (2.4)
Renal disease: PKT	6 (1.3)
Renal disease: other	3 (0.7)

All patients were aged 18 years or over. No values were missing. AKI, acute kidney injury; ESRD, end-stage renal disease; IQR, interquartile range; PKT, post-kidney transplant; SD, standard deviation.

**Table 2:** Clinical outcomes by demographic and comorbidity characteristics, with unadjusted effect measures for in-hospital mortality (*n* = 451).

Variable	Discharged Alive (*n* = 330) *n* (%)	Died (*n* = 121) *n* (%)	OR (95% CI)	*p*
Male sex	195 (59.1)	71 (58.7)	0.98 (0.64–1.50)	0.94
Saudi nationality	269 (81.5)	107 (88.4)	1.73 (0.93–3.23)	0.081
Diabetes mellitus	113 (34.2)	62 (51.2)	2.02 (1.32–3.08)	0.001 *
Hypertension	132 (40.0)	68 (56.2)	1.92 (1.26–2.93)	0.002 *
Cardiac disease	34 (10.3)	23 (19.0)	2.04 (1.15–3.64)	0.014 *
Renal disease, any	56 (17.0)	27 (22.3)	1.41 (0.84–2.35)	0.19
—ESRD	43 (13.0)	20 (16.5)	1.32 (0.74–2.35)	0.34
—AKI	6 (1.8)	5 (4.1)	2.33 (0.70–7.77)	0.17 ^‡^
—PKT/other	7 (2.1)	2 (1.7)	0.83 (0.17–4.08)	1.00 ^‡^

Reference category for each row is absence of the characteristic (female sex; non-Saudi; no comorbidity; no renal disease). * *p* < 0.05. ^‡^ Fisher’s exact test; others chi-square. Restricted to the 451 patients with determinate survival status; 6 transferred and 3 indeterminate excluded. Discharged alive includes 16 who left against medical advice. CIs by Woolf logit method; all unadjusted.

**Table 3:** Length of hospital stay by demographic and clinical characteristics (*n* = 460).

Variable	Median LOS, Days (IQR)	Comparator	*p*
Male (*n* = 271)	8 (4–16)	Female (*n* = 189): 6 (3–11)	0.006 *
Non-Saudi (*n* = 79)	9 (5–17)	Saudi (*n* = 381): 7 (3–13)	0.008 *
Diabetes (*n* = 178)	9 (5–15)	No diabetes (*n* = 282): 6 (3–13)	<0.001 *
Hypertension (*n* = 203)	9 (5–15)	No hypertension (*n* = 257): 6 (3–11)	<0.0001 *
Cardiac disease (*n* = 57)	8 (5–11)	No cardiac disease (*n* = 403): 7 (4–14)	0.50
Renal: AKI (11)/ESRD (63)/PKT (6)/other (3)/none (377)	11 (8–25)/7 (4–12)/6 (4–8)/6 (6–8)/7 (4–14)	—	0.28 ^†^

* *p* < 0.05 (Mann–Whitney U). ^†^ Kruskal–Wallis. Abbreviations as Table 1.

**Table 4:** Age and length of hospital stay (LOS) by clinical outcome.

Outcome	*n*	Age (Years) Mean ± SD	LOS (Days) Median (IQR)
Discharged alive	330	46.9 ± 19.6	6 (3–11)
Died	121	62.6 ± 17.2	11 (7–19)
Transferred	6	46.8 ± 19.4	8 (4–10)
**Overall *p*-value**		**<0.0001**	**<0.0001**

One-way ANOVA (age); Kruskal–Wallis test (LOS). IQR, interquartile range.

**Table 5:** MAI-based appropriateness of COVID-19 pharmacotherapies (*n* = 1018 prescriptions).

Drug (*n* Prescribed)	Appropriate *n* (%)	Inappropriate *n* (%)
Enoxaparin (*n* = 313)	311 (99.4)	2 (0.6)
Favipiravir (*n* = 297)	282 (94.9)	15 (5.1)
Ribavirin (*n* = 28)	26 (92.9)	2 (7.1)
Dexamethasone (*n* = 187)	172 (92.0)	15 (8.0)
Tocilizumab (*n* = 142)	113 (79.6)	29 (20.4)
Lopinavir/ritonavir (*n* = 32)	23 (71.9)	9 (28.1)
Remdesivir (*n* = 5) ^a^	3 (60.0)	2 (40.0)
Hydroxychloroquine (*n* = 14) ^a^	6 (42.9)	8 (57.1)
All	936 (91.9)	82 (8.1)

Cumulative MAI ≥ 3 indicates inappropriateness; cost domain excluded. ^a^ Fewer than 20 prescriptions: exploratory, do not generalize.

**Table 6:** Distribution of MAI domain ratings (*n* = 1018 assessed prescriptions).

MAI Domain (Guiding Question)	All Prescriptions *n* (%)	Among Inappropriate (*n* = 82) *n* (%)
Indication: is the drug indicated?	85 (8.3)	58 (70.7)
Effectiveness: is it effective for the condition?	12 (1.2)	9 (11.0)
Dosage: is the dose correct?	198 (19.4)	13 (15.9)
Correct directions: are directions correct?	3 (0.3)	0 (0.0)
Practical directions: are directions practical?	3 (0.3)	1 (1.2)
Drug–drug interaction: any significant interaction?	95 (9.3)	19 (23.2)
Drug–disease interaction: any significant interaction?	2 (0.2)	0 (0.0)
Duplication: any unnecessary duplication?	15 (1.5)	7 (8.5)
Duration: is treatment duration acceptable?	183 (18.0)	68 (82.9)
Total ratings	596	

“Flagged” = domain rated marginally appropriate or inappropriate (B or C). Cost domain excluded. A prescription may be flagged in more than one domain, so percentages do not sum to 100.

## Data Availability

All original data supporting the findings of this study are available from the corresponding author upon reasonable request.

## Abbreviations

The following abbreviations are used in this manuscript:

AKI	Acute kidney injury
APACHE II	Acute Physiology and Chronic Health Evaluation II
ARDS	Acute respiratory distress syndrome
CI	Confidence interval
COVID-19	Coronavirus disease 2019
CRP	C-reactive protein
EMR	Electronic medical record
ESRD	End-stage renal disease
ICU	Intensive care unit
IQR	Interquartile range
IRB	Institutional Review Board
LMWH	Low-molecular-weight heparin
LOS	Length of stay
MAI	Medication Appropriateness Index
MOH	Ministry of Health
OR	Odds ratio
PKT	Post-kidney transplant
RCT	Randomized controlled trial
RT-PCR	Real-time reverse transcription polymerase chain reaction
SARS-CoV-2	Severe acute respiratory syndrome coronavirus 2
SD	Standard deviation
SOFA	Sequential Organ Failure Assessment
WHO	World Health Organization
